# CHOICE MODERATES RELATIONSHIPS BETWEEN LEVEL AND DURATION OF CARE AND THE HEALTH IMPACT OF CARING FOR AN OLDER PARENT

**DOI:** 10.1093/geroni/igz038.405

**Published:** 2019-11-08

**Authors:** Robin Tarter, Dena Hassouneh, Allison Lindauer, Nathan Dieckmann

**Affiliations:** 1 Oregon Health and Science University, Portland, Oregon, United States; 2 Oregon Health & Science University School of Nursing, Portland, Oregon, United States

## Abstract

The perception of choice in the caregiving role has emerged as a key theme in qualitative gerontological caregiving research but few studies have examined choice quantitatively. The aim of our study was to test whether perceived choice moderated the relation between level and duration of care and the health impact of caring for a parent over the age of 65. We tested these questions in a series of structural equation models using existing data from the National Alliance for Caregiving, Caregiving in the U.S. 2015 Survey. We found that for adult-child caregivers of parents who reported a lack of choice in taking on the caregiving role, greater responsibilities for assistance with activities of daily living (ADLs) (p<0.01) and instrumental ADLS (p<0.01), and greater time providing care (p<0.05) predicted the negative impact of caregiving on caregiver health. The number of ADLs performed also predicted the emotional stress of caregiving for parents (p<0.01). Conversely, for caregivers who reported that they did have a choice in taking on the caregiving role, level and duration of care were not significantly related to the impact of caregiving on caregiver health, or the emotional stress of caregiving. Women were significantly more likely to report a lack of choice than men (p<0.05). Additional research is needed to explore the meaning of choice, and the ways in which choice may be especially constrained for daughters who care for older adults, in order to develop interventions to ameliorate the potentially deleterious health effects of caregiving on adult-children.

